# Neuroprotective Treatment of Postanoxic Encephalopathy: A Review of Clinical Evidence

**DOI:** 10.3389/fneur.2021.614698

**Published:** 2021-02-18

**Authors:** Sjoukje Nutma, Joost le Feber, Jeannette Hofmeijer

**Affiliations:** ^1^Department of Neurology, Medisch Spectrum Twente, Enschede, Netherlands; ^2^Clinical Neurophysiology, University of Twente, Enschede, Netherlands; ^3^Department of Neurology, Rijnstate Hospital Arnhem, Arnhem, Netherlands

**Keywords:** postanoxic coma, post-cardiac arrest syndrome, resuscitation, cerebral ischaemia, hypoxic ischaemic brain injury

## Abstract

Postanoxic encephalopathy is the key determinant of death or disability after successful cardiopulmonary resuscitation. Animal studies have provided proof-of-principle evidence of efficacy of divergent classes of neuroprotective treatments to promote brain recovery. However, apart from targeted temperature management (TTM), neuroprotective treatments are not included in current care of patients with postanoxic encephalopathy after cardiac arrest. We aimed to review the clinical evidence of efficacy of neuroprotective strategies to improve recovery of comatose patients after cardiac arrest and to propose future directions. We performed a systematic search of the literature to identify prospective, comparative clinical trials on interventions to improve neurological outcome of comatose patients after cardiac arrest. We included 53 studies on 21 interventions. None showed unequivocal benefit. TTM at 33 or 36°C and adrenaline (epinephrine) are studied most, followed by xenon, erythropoietin, and calcium antagonists. Lack of efficacy is associated with heterogeneity of patient groups and limited specificity of outcome measures. Ongoing and future trials will benefit from systematic collection of measures of baseline encephalopathy and sufficiently powered predefined subgroup analyses. Outcome measurement should include comprehensive neuropsychological follow-up, to show treatment effects that are not detectable by gross measures of functional recovery. To enhance translation from animal models to patients, studies under experimental conditions should adhere to strict methodological and publication guidelines.

## Introduction

Survival rates of out-of-hospital cardiac arrest have increased considerably over the past decades (1, 2). In contrast, neurological outcome of survivors has improved only marginally. Of patients surviving up to hospital admission, more than three quarters initially remain comatose as a result of diffuse cerebral ischaemia (3, 4). Comatose patients after circulatory arrest are treated on intensive care units to support airway, breathing, and circulation. Anoxic-ischemic encephalopathy is the key determinant of death and disability, with rates of in-hospital mortality or enduring neurologic impairment >50% (5). Targeted temperature management (TTM) at 33 or 36°C is applied as a therapeutic strategy in most hospitals to improve brain recovery, although the clinical evidence supporting efficacy is complex and mechanisms of action are incompletely understood (6–9).

Treatment strategies other than TTM that were beneficial in animal models and have been tested in clinical trials include glutamate and calcium antagonists, anti-inflammatory therapies, and anti-oxidants. None of these improved cerebral recovery or functional outcome of patients after cardiac arrest. Proposed explanations include poor extrapolation from animal models to patients, insufficient knowledge of when and where we can interfere in the complex pathophysiology of brain damage after global ischaemia, and heterogeneity of patients groups (10, 11).

In this article, we review the clinical evidence of efficacy of neuroprotective treatments in comatose patients after cardiac arrest. We discuss treatment effects, and the lack thereof, in the context of the pathophysiology of postanoxic encephalopathy, and propose future directions.

## Search Strategy and Selection Criteria

**Figure 1:**
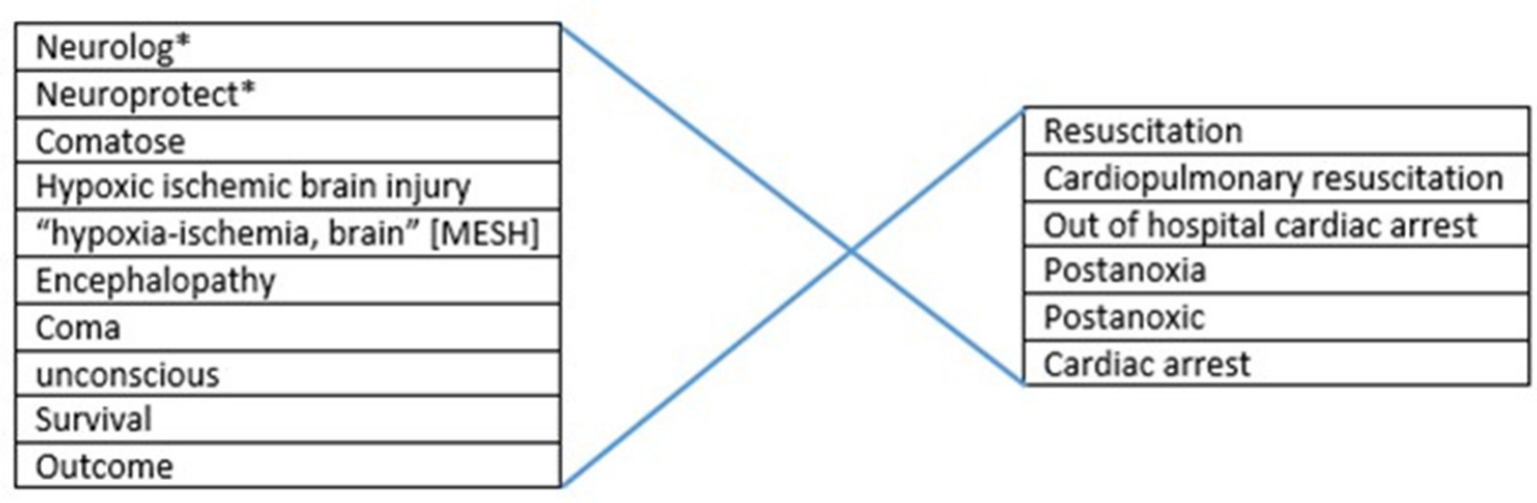
Overview of used search strategy.

For analysis of tested neuroprotective measures in comatose patients after out-of-hospital cardiac arrest (OHCA), we applied a search in the Medline and Pubmed databases until October 2019. We used several combinations of the keywords and MeSH terms. For selection of the target patient group we used “post-anoxic encephalopathy,” “hypoxic ischemic brain injury,” “coma,” “cardiopulmonary resuscitation,” and “cardiac arrest” (Figure 1). For selection of interventions we first used the general term “neuroprotective” in combination with “outcome,” later we searched more specifically on tested interventions such as “xenon,” “magnesium” etc. Review articles were used to screen reference lists. We included prospective, controlled, intervention trials with clinical neurological outcome measures. Studies that used the cerebral biomarkers NSE or S100b as a substitute for neurological outcome were also included. Technique of cardiopulmonary resuscitation was not taken into account as an in- or exclusion criterion. When a certain topic was addressed by a large number of studies, the latest meta-analysis was used, supplemented by more recent studies in that field. This applied to hypothermia and adrenaline (epinephrine). We excluded studies on in-hospital cardiac arrest, retrospective, observational, and uncontrolled studies, case studies, studies in pediatric populations, and animal studies.

## Results

**Figure 2:**
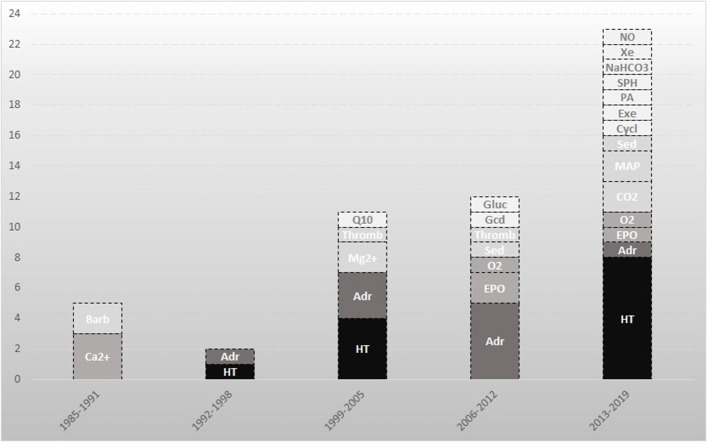
Neuroprotective studies directed at improvement of neurological outcome after cardiac arrest. An overview of the included studies in this review. The darker the box, the larger the amount of included studies on that topic. Barb indicates barbiturates; Ca^2+^, calcium antagonists; CO_2_, carbon dioxide; Cycl, cyclosporine; Adr, adrenaline; EPO, erythropoietin; Exe, exenatide; Gcd, glucocorticoid; Gluc, glucose; HT, hypothermia; Mg^2+^, magnesium; MAP, mean arterial pressure; NaHCO_3_, sodium bicarbonate; NO, Sodium nitrite; O2, oxygen; PA, prophylactic antibiotics; Q10, coenzyme Q10; Sed, sedation; SPH, scopolamine and penehyclidine hydrochloride; Thromb, thrombolysis; Xe, xenon.

We included 53 studies on 21 different therapies. Over the past 30 years there has been a significant increase in trials on cerebral recovery after resuscitation (Figure 2). The first years were characterized by studying the effects of barbiturates, calcium antagonists, and hypothermia. Later, the focus shifted to novel therapies in this field like xenon, exenatide, and cyclosporine.

Since we included a multitude of therapies, addressed in studies with disparate diagnostic criteria and study designs, meta-analysis was not possible. Therefore, a narrative review was chosen. We found two cohort studies on treatment of status epilepticus, but no prospective controlled studies. A randomized controlled trial is currently in progress (12). Several randomized controlled trials (RCT) on early application of extracorporeal life support in cardiac arrest are in progress [NCT03065647 (13), NCT01605409 (14), NCT02527031 (15), NCT01511666 (16), NCT03101787 (17)], no results are published yet. Also no studies could be found concerning specific nutrition after cardiac arrest. Study details and results are summarized in Supplementary Table 1.

## Pathophysiological Considerations

**Figure 3:**
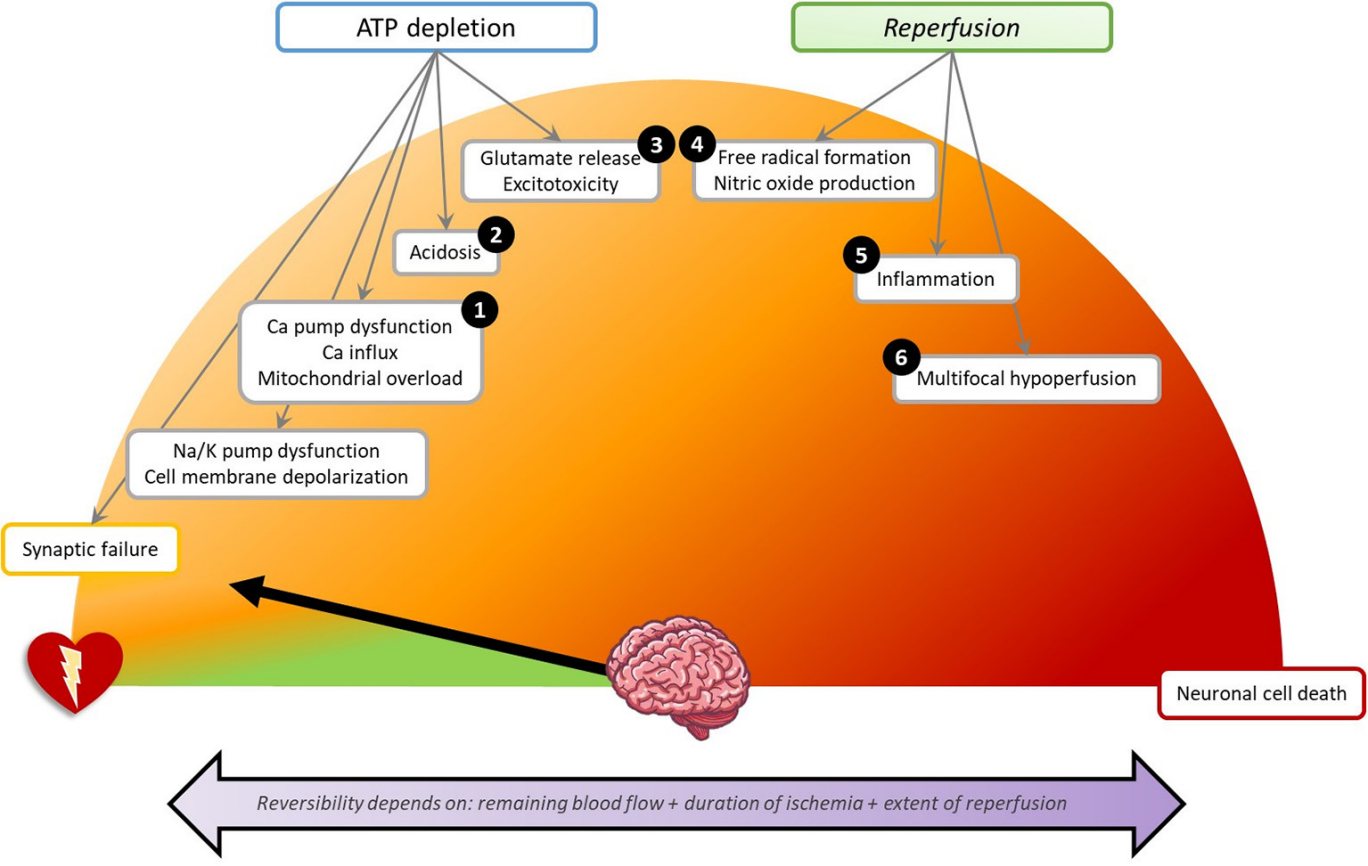
Schematic overview of pathophysiology of brain damage in the first 72 h after cardiac arrest. Each step can lead to direct or delayed neuronal cell death. The numbers indicate the presumed point of action of the discussed neuroprotective treatments. (1) Calcium antagonists: Nimodipine, Flunarizine, Lidoflazine. Mitigating mitochondrial damage: Cyclosporine, Coenzyme Q10. (2) Preventing acidosis: Sodium bicarbonate. (3) Glutamate antagonism: Noble gases, Exenatide, Scopolamine, and penehyclidine hydrochloride, Magnesium. (4) Antioxidants: Preventing hyperoxia, Sodium nitrite. (5) Anti-inflammation: Erythropoietin, Glucocorticoids. (6) Optimizing cerebral perfusion: Adrenaline, Mild hypocapnia, High mean arterial pressure, Thrombolysis. (1–6) Pan-inhibition: Hypothermia. Not indicated by a number: Decreasing cerebral metabolism: Barbiturates. Supportive therapies: Sedation, Glucose regulation, Prophylactic antibiotics. Na/K, sodium/potassium; Ca, calcium.

The human brain contributes only 2% to the total body weight, yet it accounts for 20% of total body oxygen consumption and 25% of glucose utilization (18, 19). This high metabolism in combination with the lack of cerebral glucose stores makes the brain highly susceptible to blood flow interruption. Insufficient blood flow (ischaemia) and oxygen delivery (hypoxia) during cardiac arrest may lead to loss of neuronal function within seconds (20). Initially, this is reversible. Transitions from reversible to irreversible brain damage occur in minutes, hours, or days, and depend on the level of the remaining blood flow, the duration of ischaemia, and the extent of reperfusion (Figure 3) (21).

Disappearance of synaptic activity is the first effect of cerebral hypoxia or ischaemia and occurs within seconds to minutes (22). Synaptic activity indicates the process in which neurons pass chemical signals to other neurons and is also called neurotransmission. Early synaptic failure is due to impaired presynaptic neurotransmitter release (11, 23). Synaptic failure may be completely reversible. However, with lasting hypoxia or ischaemia, synaptic disturbances may become permanent, even with preserved membrane potential (24).

Depending on the remaining perfusion levels, cerebral glucose and ATP stores are depleted within minutes to hours (19). Ultimately, this results in dysfunctioning of ATP-dependent ion pumps, especially transmembrane sodium-potassium pumps. The subsequent inflow of sodium and outflow of potassium leads to loss of ion gradients across the plasma membrane, causing depolarization (i.e., loss of membrane potential) (19). This leads to inability to generate action potentials. Since the net flow of osmotically active particles from the extracellular space into the neurons (sodium, chloride) exceeds that from intracellular to extracellular (potassium), the intracellular osmolality increases. This causes inflow of water into the cells leading to cell swelling (25). Cell swelling is reversible with rapid restoration of perfusion. In the absence of reperfusion, it leads to necrotic cell death.

Dysfunctioning of ATP-dependent calcium channels causes influx of calcium into the intracellular space, which activates pathways leading up to apoptosis (19). High intracellular calcium activates the mitochondrial permeability transition pore. This protein in the inner membrane of mitochondria only opens under pathological conditions and releases cytochrome C, an activator of various cascades leading to apoptosis (26, 27) Very high calcium leads to direct destruction of mitochondria (27). In addition, calcium mediates release of glutamate, resulting in overexcitation of the NMDA receptor (“excitotoxicity”), leading to neuronal damage and cell death (28, 29).

Restoration of perfusion may cause additional (secondary) brain damage. First, free radical or reactive oxygen species may cause cellular lipid and protein degradation. Second, reperfusion is associated with inflammatory responses and microvascular damage (19). Third, reperfusion is often unevenly distributed due to cerebral vasospasm, increased blood viscosity, and platelet aggregation. This causes focal or multifocal hypoperfusion, which is called “no-reflow phenomenon” (30).

The timescales of the various pathophysiological processes define the therapeutic windows of opportunity for neuroprotective treatments interacting with these processes. The first minutes to hours after diffuse cerebral ischaemia by cardiac arrest are characterized by massive cortical synaptic failure. This is reflected by suppressed EEG patterns. With timely reperfusion this is, in principle, reversible. However, synaptic failure lasting over 6–24 h is associated with transitions toward permanent brain damage (31, 32). With deep or persistent hypoperfusion, cell swelling occurs, which is reversible with rapid restoration of perfusion, but may lead to cell death within minutes (33). Pathways leading up to programmed cell death (apoptosis) are activated on timescales up to 72 h after cardiac arrest (34). This suggests that time windows of opportunity for interventions to prevent the transition from reversible to irreversible brain damage lie between hours and days, with largest effects in the first hours after cardiac arrest (20). Interventions to improve brain network and functional recovery by enhancement of connectivity may be effective on longer timescales (35).

## Measuring Neurological Outcome

### Clinical Outcome Scales

**Figure 4:**
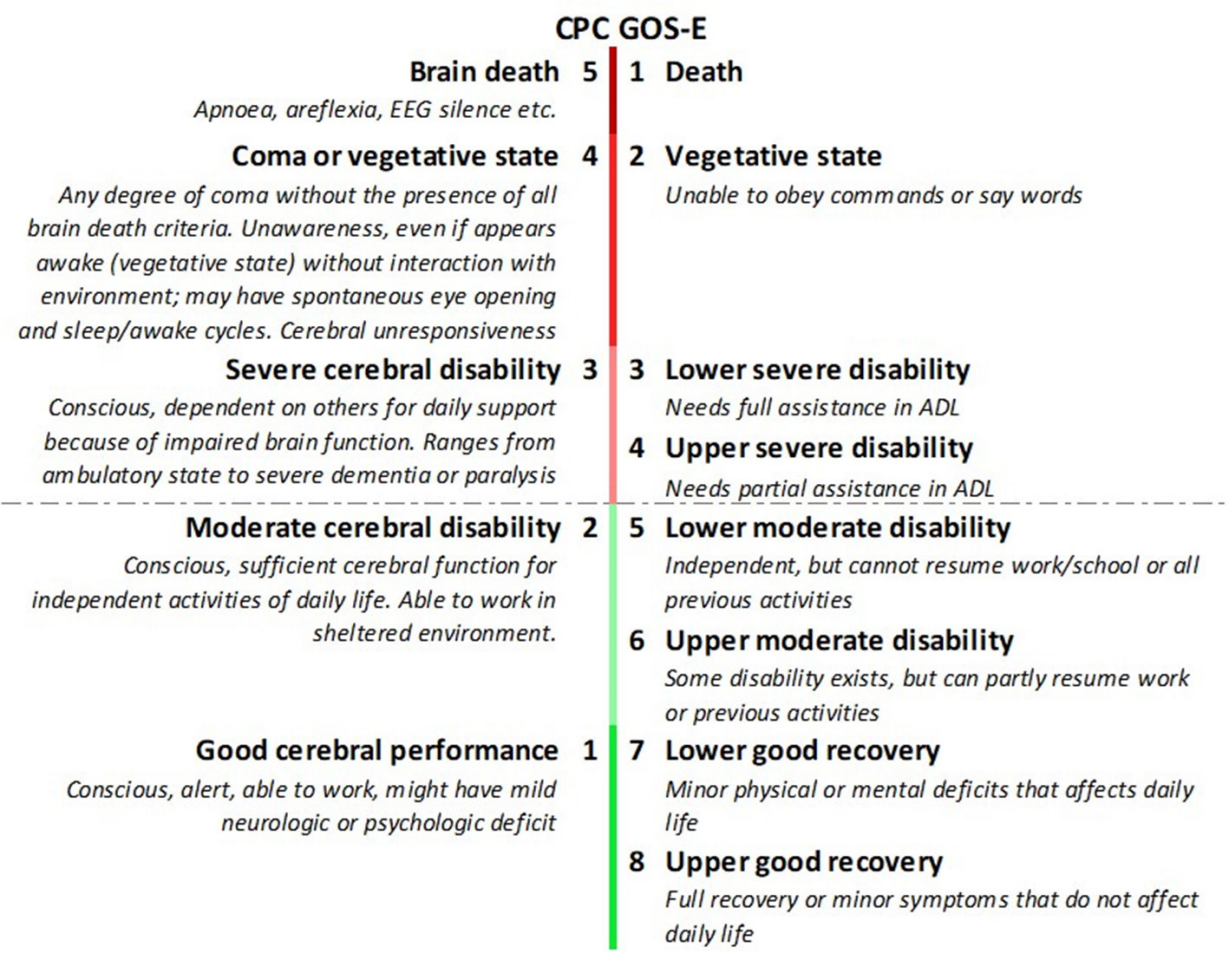
Cerebral Performance Category (CPC) compared to extended Glasgow Outcome Scale (GOS-E). CPC 1-2 and GOS-E 5-8 are considered good neurological outcome.

We included studies that used functional recovery according to the Cerebral Perfomance Category (CPC) (36) or the extended version of the Glasgow Outcome Scale (GOS-E) (37) as measures of outcome. CPC is a five-point scale ranging from brain death (CPC 5) to full recovery or mild disability (CPC 1). CPC 1–2 is mostly considered as good and CPC 3–5 as poor neurological outcome. The GOS-E uses 8 different levels of disability in which a score of 1 equals death and 8 good recovery. A score of 5–8 is considered good neurological outcome (Figure 4). Both outcome scales are criticized for limited discrimination (38, 39) and GOS-E is only validated in patients with traumatic (and not in anoxic) brain injury (40, 41).

### Biochemical Biomarkers

We also included studies that used blood levels of neuron specific enolase (NSE) or S-100b as measures of outcome. NSE and S-100b are proteins that are released in the blood and cerebrospinal fluid with damage of neurons and glial cells, respectively (42). Higher NSE blood values at day 1 and 3 are associated with poorer outcome after out-of-hospital cardiac arrest (43, 44). Reported cut-off values for reliable prediction of poor outcome range from 20 to 65 μg/L (45). Higher S-100b blood values are associated with poorer neurological outcome, but the predictive values for individual patients are limited (46). S-100b is also present in muscle and adipose tissue and can therefore be increased by a thoracic trauma due to cardiopulmonary resuscitation (47). Because of the extra-cerebral sources, heterogeneous measurement techniques, and divergent proposed cut-off threshold for prediction, the use of these biomarkers for prediction of outcome after cardiac arrest is controversial (45–47).

## Neuroprotective Treatments of Comatose Patients After Cardiac Arrest

### Pan-Inhibition

#### Hypothermia

The working mechanism of induced hypothermia is presumed to be pan-inhibition, by reducing ATP depletion (7, 48) and anoxic depolarization (8, 49). Also glutamate antagonism (50, 51), anti-inflammatory effects (52, 53), reduction of free radical production (9) and anti-apoptotic effects (54, 55) have been described. In 2002, in two relatively small RCT's (*n* = 77 and *n* = 273), TTM at 32–34°C was associated with a higher probability of a good outcome of comatose patients after cardiac arrest (respectively, 21/43 vs. 9/34 and 75/136 vs. 54/137) (56, 57). However, control groups contained relatively many patients with hyperthermia which is associated with secondary damage after an anoxic insult (58). This led to the believe that the beneficial factor was not the induced hypothermia, but the prevention of fever. The Targeted Temperature Management trial (TTM) compared controlled normothermia at 36°C with hypothermia at 33°C for 28 h and showed no significant differences in mortality (50% in 33°C group vs. 48% in 36 °C) and no differences in neurological outcome measured by CPC (CPC 1–2 46.5 vs. 47.8%) (6).

Several trials focussed on timing of inducing hypothermia and alternative cooling techniques. A 2018 RCT on alternative cooling techniques (59) showed no differences between the different techniques, but did find a significantly better survival in the intervention compared to historical controls receiving normothermia. Information on incidence of hyperthermia in this group, was lacking. A Cochrane review and later published trials comparing pre-hospital and in-hospital start of cooling also showed no certain benefit of a pre-hospital start for survival or neurological outcome (60–63), and even showed higher rates of pulmonary edema when rapid infusion of cold saline fluids were used for induction of cooling (63). A study on longer duration of hypothermia at 33°C also showed no differences in outcome (64), but did report on impaired thrombin without an increase of bleeding complications (65). The ongoing TTM2 trial compares target temperature of 33°C and standard care avoiding fever [NCT02908308 (66)]. If beneficial at all, optimal timing and duration of targeted temperature management remain to be elucidated.

The association of decreased mortality when using neuromuscular blockade during hypothermia (67–69), could not be confirmed by two small RCT's comparing hypothermia treatment with and without neuromuscular blockade (70, 71).

### Decreasing Cerebral Metabolism

#### Barbiturates

After several feasibility trials using barbiturates in comatose patients after cardiac arrest (72), these substances were tested in efficacy trials on neuroprotection after cardiac arrest in the 1980s. The presumed working mechanism involves global depression of cerebral metabolism (73), depression of release of ROS, and inhibition of lipid degradation (74, 75). A randomized trial tested a single thiopental dose in comatose patients after cardiac arrest and found no significant effects on survival (77% in intervention group vs. 80% in standard-therapy group) or in good cerebral recovery (20 vs. 15%) (76). A smaller study with a control group of matched historical cases also found no differences in survival, despite a non-significant higher mortality in the first 6 h in the thiopental group in patients with ischemic heart disease. However, after these 6 h, good neurological recovery was observed significantly more frequent in the thiopental group (61 vs. 37%, *p* < 0.03) (77).

### Glutamate Antagonism

#### (Noble) Gases

Various *in vitro* and animal studies showed beneficial effects of noble gases on hypoxic-ischemic brain damage (78, 79). This resulted in feasibility and safety studies on xenon, helium, and hydrogen in patients with postanoxic encephalopathy after cardiac arrest (80–82). The presumed mechanism of action is competitive inhibition at the glycine co-activation site of the NMDA receptor, thereby preventing toxic overexcitation. A single-blinded, randomized study in 110 patients studied the effect of inhaled xenon on white matter damage assessed by diffusion tensor magnetic resonance imaging (MRI). Fractional anisotropy was higher in the intervention than in the control group, suggesting less damage of white matter tracts. However, functional recovery as expressed by CPC and mRS scores at 6 months showed no differences between the groups (83). An RCT on the effects of inhalation of hydrogen on neurological outcome is in progress [UMIN000019820 (84)].

#### Exenatide

The glucagon-like peptide-1 (GLP-1) exenatide is used for treatment of type 2 diabetes mellitus. It showed neuroprotective and anti-inflammatory capacities in several *in vivo* and *in vitro* studies (85). Exenatide is a mediator of glutamate release and can prevent toxic over-excitation by inhibiting glutamate release (86, 87). Exenatide given in the first 6 h after return of spontaneous circulation (ROSC) had no statistically significant effect on NSE levels or clinical outcome in an RCT with 118 patients (88).

#### Scopolamine and Penehyclidine Hydrochloride

Another possible therapy targeting the NMDA-receptor is penehyclidine hydrochloride (PHC). In a study with 80 patients randomized to either scopolamine or PHC, PHC was associated with lower intracranial pressures, higher cerebral perfusion pressures and lower NSE. However, clinical outcome measures and a control group without experimental treatment were lacking, hampering interpretation of the data (89).

#### Magnesium

*In vitro* and *in vivo* studies showed beneficial effects of magnesium on neuronal and neurological recovery after hypoxic-ischemic damage, due to reduction in glutamate response and calcium entry blocking capacities. Two randomized placebo controlled studies focussed on the effects of magnesium sulfate, both in a pre-hospital setting. The first (*n* = 105), used magnesium in patients with refractory ventricular fibrillation and showed no differences in gaining ROSC or in neurological outcome of the three surviving patients (90). The second (*n* = 300), studied effects of diazepam with or without magnesium. Neurological outcome expressed as awakening and having comprehensible speech was not significantly different between both groups (91). Other studies on magnesium in cardiac arrest did not address neurological recovery or were focussed on in-hospital cardiac arrest.

### Calcium Antagonists

#### Nimodipine

Of all the calcium antagonists nimodipine is studied most in comatose patients with postanoxic encephalopathy. After a safety study of nimodipine in 22 OHCA patients in 1987 (92), a randomized double-blind study in 155 patients administered nimodipine or placebo in a prehospital setting. No effects were demonstrated on long term survival or probability of good neurological outcome (93). Another randomized study in 51 patients showed significantly higher cerebral blood flow in the nimodipine group, without differences in intracranial pressure measured on several time points, compared to placebo. Neurological outcome did not differ between the groups (94). A smaller randomized trial measured the intracranial pressure continuously and found lower mean pressures in the nimodipine group. Neurological outcome nor survival was taken into account (95).

#### Lidoflazine

The Brain Resuscitation Clinical Trial II Study Group included 520 comatose patients after cardiac arrest and randomized them to lidoflazine or placebo. At 6 months there was no difference in mortality (82% in lidoflazine group vs. 83% in placebo group), or in proportion of patients with good outcome (15 vs. 13%) (96).

### Preventing Acidosis

#### Sodium Bicarbonate

Sodium bicarbonate (NaHCO_3_) has been used to reverse acidosis and treat hyperkalaemia in cardiopulmonary resuscitation. Both favorable and unfavorable effects of administering NaHCO_3_ during CPR have been reported. Unfavorable effects include paradoxical respiratory acidosis due to increased carbon dioxide tension (97). Observational studies showed a possible role for NaHCO_3_ in prolonged cardiopulmonary resuscitation (CPR), to compensate the consequent severe acidosis that is associated with an impaired responsiveness to catecholamines (98). A large randomized study on prehospital NaHCO_3_ administration (*n* = 792) found no differences in survival (13.8% vs. 13.9%), but confirmed a possible beneficial effect in prolonged CPR with a trend toward increased survival (32.8% in the intervention group vs. 15.4%, *p* = 0.07) (99). The latest RCT in 50 patients focussed only on prolonged cardiac arrest (>10 min) and treated with NaHCO_3_ or placebo when there was evidence of severe metabolic acidosis. Despite a significant difference in pH (6.99 in intervention group vs. 6.9 in placebo group) there were no differences in survival and neurological outcome (100).

### Antioxidants

#### Preventing Hyperoxia

Animal studies demonstrated that hyperoxia in the first 24 h after cardiac arrest is associated with poor neurological outcome (101). A prospective observational study on patients after cardiac arrest showed an independent association between early hyperoxia and poor neurological outcome (102). Four studies addressed the feasibility of lowering oxygen levels in a pre-hospital setting (103–106). Three of the four studies showed feasibility. The biggest risk was inadvertent hypoxaemia (103, 105). The groups were small and often neurological outcome measures were not taken into account, so conclusions on efficacy cannot be drawn. Another study on early reduction of oxygen levels post cardiac arrest is in progress (NCT03138005) (107). A larger study by the COMACARE study group compared systemic arterial normoxia (PaO_2_ 10–15 kPa) to moderate hyperoxia (PaO_2_ 20–25 kPa) and found no significant differences in the primary endpoint NSE serum concentration at 48 h after cardiac arrest or in neurological outcome measured by CPC (108).

#### Sodium Nitrite

During hypoxia nitrite is converted to nitric oxide via several pathways (109). This free radical has shown to be neuroprotective by reducing production of reactive oxygen species in animal studies (110, 111). A pilot study intravenously administered sodium nitrite to 120 patients during resuscitation from OHCA. Due to insufficient serum levels, the dose of 25 mg was halfway adjusted to 60 mg. Despite adequate serum levels, there were no differences in rate to ROSC, survival or neurological outcome at discharge compared to matched controls. There were no differences in systolic blood pressure or use of noradrenaline (norepinephrine) between both groups (112).

### Anti-inflammation

#### Erythropoietin

Promising pre-clinical studies suggested neuroprotective effects of erythropoietin (EPO) by inhibition of neuronal apoptosis (113) and anti-inflammatory qualities (114). This gave rise to three clinical studies. A prospective study with case matched controls (*n* = 58) showed no significant difference in survival on day 28. One case of arterial vascular thrombosis was observed as adverse event in the EPO-group (115). The second study (*n* = 72) also used case matched controls and found a significantly higher rate of survival up to hospital admission and 24h survival in the intervention group (92 vs. 65% and 83 vs. 52%), but no difference in CPC at hospital discharge (116). The third and largest study was an RCT in 476 patients and found no significant difference in CPC-scores at any time point. Serious adverse events consisting of thrombotic complications, were more frequent in the EPO-treated group (117).

#### Glucocorticoids

Since cardiac arrest is associated with an impaired cortisol release from the adrenal cortex, two clinical trials focused on the effects of glucocorticoid suppletion on outcome. A pilot study compared hydrocortisone with placebo during resuscitation, resulting in a large increase in attaining ROSC in the hydrocortisone group (61 vs. 39%, *p* = 0.038), but a comparable median CPC of 4 at discharge in four surviving patients of each group (118). Other studies on glucocorticoid administration focussed on in-hospital cardiac arrests (119).

### Mitigating Mitochondrial Damage

#### Cyclosporine

This immunosuppressant substance used in the treatment of for example Crohn's disease got new attention when *in vitro* and *in vivo* studies showed promising effects on preventing mitochondrial permeability (120). In an RCT in 794 patients, cyclosporine was administered during resuscitation of non-shockable out-of-hospital cardiac arrest. Survival, neurological outcome, and the primary endpoint of multi-organ failure were essentially the same in the two groups (121).

#### Coenzyme Q10

(CoQ10) is an electron transporter between mitochondrial complexes. Dysfunction compromises mitochondrial function. Administration of CoQ10 has shown neuroprotective effects in for example Parkinson's disease (122). One observational study showed an association between low CoQ10 levels and increased mortality after cardiac arrest (123). An RCT in 49 patients compared CoQ10 suppletion with placebo within 6 h after cardiac arrest during 5 days, survival at 3 months was significantly increased in the CoQ10 group (17 of 25 vs. 7 of 24 patients). Persistent vegetative state was more frequent in the CoQ10 group (7 vs. 3 patients) (124).

### Optimizing Cerebral Perfusion

#### Adrenaline

Adrenaline has been an established medicine in advanced life support protocols for many years (125). By stimulating the α-adrenergic receptors it causes vasoconstriction and thereby a higher coronary blood flow and a bigger chance of ROSC after cardiac arrest (126). A positive effect on neurological outcome was never found, which has been attributed to platelet activation mediated by adrenaline induced thrombosis, with impairment of cerebral blood flow (127). A large randomized trial (*n* = 8,014) compared adrenaline to placebo and found significantly higher survival rates at 30 days, but worse neurological functioning in the surviving intervention group (128). A recent meta-analysis concluded that a standard dose of adrenaline compared to pooled treatments (defined as placebo, no drugs, high dose of adrenaline or adrenaline + vasopressin) improves survival to hospital and increases the chances of a good neurological outcome. However, when standard dosed adrenaline was compared with just placebo or no drugs, no significant differences in neurological outcome were found (129). It can be concluded that optimal dosing and effects on neurological outcome are still unclear.

#### Carbon Dioxide Levels

Mild hypercapnia should compensate the compromised cerebral blood flow after cardiac arrest by augmenting cerebral perfusion due to vasodilation (130). However, higher carbon dioxide levels carry the risk of increased intracranial pressure and of pulmonary vasoconstriction. On the other hand, hypocapnia is associated with worse neurological outcomes (131). Probably, preventing hypocapnia and inducing mild hypercapnia is beneficial. A feasibility trial in 83 patients compared normocapnia (PaCO2 35–45 mmHg) with mild hypercapnia (PaCO_2_ 50–55 mmHg) and found no differences in GOS. The increase in NSE was significantly lower in the hypercapnia group 24, 48, and 72 h (132). A large RCT comparing these same PaCO_2_ levels (TAME) is in progress (133). Another RCT (*n* = 123) compared low-normal PaCO2 (33–35 mmHg) with high-normal PaCO_2_ (43–45 mmHg), maintained this for 36 h. There were no significant differences in NSE at 48 h or in neurological outcome. In the high-normal PaCO_2_ group there was one case of unexplained cerebral oedema on CT scanning. Two patients had severe ARDS (108).

#### Mean Arterial Pressure

Many observational studies showed an association between a higher mean arterial pressure (MAP) and an increase in survival and improvement in neurological outcome (134). On the other hand vasoactive medication is associated with increased mortality (135). The first prospective trial on this topic dates from 2018 and compared low-normal (65–75 mmHg) to high normal (80–100 mmHg) MAP maintained for 36 h after cardiac arrest in 120 patients. There were no significant differences in the primary outcome measure of NSE at 48 h, nor in neurological outcome (136). A more recent study randomized 112 post-cardiac arrest patients to a protocol focussed on haemodynamic optimization (MAP 85–100 mmHg and SvO2 65–75%) or a MAP of 65 mmHg. Their primary outcome measure, cerebral damage according to DW-MRI, showed no differences between the two groups. Neurological outcome at discharge and after 6 months was the same in both groups (137).

#### Thrombolysis

To target microthrombi involved in the no-reflow phenomenon, fibrinolytic therapy has been studied to ameliorate cerebral damage. Studies in humans were often ambivalent on the point of action of thrombolysis, applying thrombolysis mainly for resolving pulmonary embolism or coronary thrombosis, and not primarily to improve cerebral blood flow. After some feasibility studies without data on neurological outcome (138, 139), a long term follow-up study in a small population suggested beneficial effects of thrombolytic therapy on neurological outcome (140). The first randomized controlled trial studied the effects of thrombolysis in patients with pulseless electrical activity (PEA). Of the 233 included patients only one survived (141), so no conclusions on effects on neurological outcome can be drawn. A larger trial enrolled 1,050 patients with a witnessed arrest of presumed cardiac origin. At 30 days there was no differences in survival (77 in thrombolysis group, 89 in placebo group) or neurological outcome. Intracranial hemorrhage occurred more often in the intervention group (14 vs. 2 patients) (142). Guidelines on cardiac arrest treatment now state that thrombolysis should only be considered in case of suspected pulmonary embolism (4).

### Supportive Therapies

#### Sedation

Several studies addressed sedation techniques targeting rapid awakening after discontinuation of sedation in comatose patients after cardiac arrest. Two prospective studies took neurological outcome into account as a secondary endpoint. The first randomized study (*n* = 59) compared propofol/remifentanil (PR) with midazolam/fentanyl (MF) and concluded that the time to extubation was significantly shorter in the PR group. There was no difference in neurological outcome (143). A later cohort study compared two sedation regimens used in different time blocks (2008–2013 vs. 2014–2016) and also found a smaller delay in awakening in the PR group, with no significant changes in good neurological outcome (144). No studies compared sedation with no sedation in this population.

#### Glucose Regulation

Large fluctuations in blood glucose levels and hyperglycaemia are associated with poor neurological outcome and death in comatose patients after cardiac arrest (145). In accordance with studies in critically ill patients in general, maintenance of normoglycemia is advised in patients after cardiac arrest. In an RCT in 90 patients two different glucose regulation regimes where compared, with no differences in mortality between strict (blood glucose of 4–6 mmol/L) vs. moderate (blood glucose of 6–8 mmol/L) glucose control (146).

#### Prophylactic Antibiotics

A pilot trial compared prophylactic vs. clinically-driven treatment with antibiotics after cardiac arrest. The main hypothesis was that prevention of early onset pneumonia should decrease the severity of the systemic inflammatory response after resuscitation. There was no significant difference in survival or neurological outcome (147). The results of another trial on antibiotherapy to prevent infectious complications after cardiac arrest are still pending [NCT02186951 (148)].

## Discussion

None of the neuroprotective treatments that effectively reduced brain damage after global cerebral ischemia in animal models improved outcome of patients with postanoxic encephalopathy after cardiac arrest in clinical trials, unequivocally. This includes TTM at 33 or 36°C (6). Although, compelling evidence shows that hyperthermia is associated with poor neurological outcome (58, 149, 150), the evidence of efficacy of lowering brain temperature to 32–34°C is complex.

An important limitation of previous and ongoing trials on neuroprotective treatments after cardiac arrest is the lack of subgroup analyses according to measures of encephalopathy severity. It is unlikely that the divergent pathophysiological scenarios ranging from reversible synaptic failure to severe cell swelling and inflammation all warrant the same neuroprotective strategy. International guidelines on treatment of comatose patients after cardiac arrest recognize that “whether certain subpopulations may benefit from lower or higher temperatures remains unclear” (4). To fill that knowledge gap, previous and ongoing clinical trials, such as TTM2 [NCT02908308 (66)] and TAME [NCT03114033 (133)], include predefined subgroup analyses according to widely-accepted factors, such as reflow times, causes of arrest, and initial cardiac arrest rhythm. Although relevant, these are mostly indirect indicators of encephalopathy severity.

A recent analysis of 1,319 comatose patients after cardiac arrest demonstrated divergent effects of TTM at 33°C in mild vs. severe encephalopathy both with and without cardiopulmonary failure (151). This is supported by experimental studies in animal models, showing interaction between cooling and severity of encephalopathy (152, 153). Over the past decade, a multitude of studies on outcome prediction of comatose patients after cardiac arrest have identified reliable and easily retrievable direct measures of encephalopathy severity, such as EEG (32, 154), imaging (155) and biochemical measures (44). Systematic collection of such measures at baseline, with sufficiently powered predefined subgroup analyses, provides an opportunity to identify treatment effects in relatively homogeneous subgroups of patients with postanoxic encephalopathy.

Another factor hampering detection of treatment effects after cardiac arrest is the choice of outcome measures. Traditionally, for pragmatic reasons, 5 or 6 point scales of functional recovery are used, such as the CPC scale or the GOS. These measure gross neurological recovery, but cannot detect small differences in cognitive or behavioral functioning. Several studies used NSE (89, 104, 108, 132, 156), like intracranial pressure (89, 94, 95), near-infrared spectroscopy (108), cerebral blood flow (94), and MRI (83, 89, 137), as a surrogate outcome measures. However, it is largely unclear how these correlate with neurological outcome. Instead of using global outcome scales or indirect parameters of cerebral damage, detailed neuropsychological testing at 6 or 12 months after cardiac arrest holds potential to detect small, but meaningful, cognitive effects of new therapies under study.

Lack of extrapolation from animal models to patients has been discussed extensively. In addition to obvious disparities between animal models and patients (157), reasons include methodological flaws of animal studies, like the lack of sample size calculations, lack of randomization, and unblinded outcome assessments (10, 158). This, in combination with a presumed large publication bias, leads to an overstatement of efficacy of at least 30% (158). To improve meaningful extrapolation from animal models to patients, experimental animal studies should adhere to methodological quality guidelines, and journals are encouraged to use strict publication criteria (159, 160)

This is a narrative review. We included all prospective, controlled, intervention trials, without a systematic analysis of the quality of the included studies and resulting evidence according to the PRISMA guidelines. A multitude of factors hampers interpretation of data. In general, populations were small and heterogeneous, without sufficient details on in-hospital treatment. Therefore, our appreciation of the evidence, and the lack thereof, is qualitative. However, previous meta-analyses of effects of hypothermia (150), adrenaline (129), or erythropoietin (161) led to essentially the same conclusions.

## Conclusion

Promising results from animal studies on neuroprotective treatments in postanoxic encephalopathy could not be extrapolated to patients after cardiac arrest. This lack of extrapolation is related to overestimation of pre-clinical evidence, and critical disparities between animal models and patients. Almost all previous studies focussed on neuronal inhibition, but brain stimulation possibly holds a larger potential to improve brain recovery after cardiac arrest. Future clinical trials should be conducted with sufficiently large, well-described populations. Outcome measurement should include comprehensive neuropsychological follow-up, to show treatment effects that are not detectable by gross measures of functional recovery.

## Author Contributions

SN: conceptualization, methodology, investigation, writing—original draft, writing—review & editing, and visualization. JF: writing—review & editing. JH: conceptualization, writing—review & editing, visualization, and supervision. All authors contributed to the article and approved the submitted version.

## Conflict of Interest

The authors declare that the research was conducted in the absence of any commercial or financial relationships that could be construed as a potential conflict of interest.
