# Supplementary material for: Neuroprotective Treatment of Postanoxic Encephalopathy: A Review of Clinical Evidence
Source: Front Neurol. 2021 Feb 18;12:614698. doi: 10.3389/fneur.2021.614698 (PMC7930064; doi:10.3389/fneur.2021.614698)
Supplement: Supplementary file 1 [file Table_1.docx]

| **Table 1: Clinical trials on neuroprotective strategies and their main results** | | | | | | | | | | | | | |
| --- | --- | --- | --- | --- | --- | --- | --- | --- | --- | --- | --- | --- | --- |
| **Therapy** | **Source** | **Year of publication** | **Study design** | **Intervention** | **n** | **Population** | **Start of intervention** | **End of intervention** | **TTM** |  |  | **Outcome measures** | **Main results** |
| *Pan-inhibition* | |  |  |  |  |  |  |  |  |  |  |  |  |
| **Hypothermia** | Bernard et al.(56) | 2002 | Randomized controlled trial, with blinded outcome assessment | hypothermia vs. no temperature regulation | 77 | OHCA due to VF | Exact time unknown, but in the ambulance | 12h active cooling stops 18h passive rewarming starts | Partly | 33˚C vs. > 37˚C |  | Good outcome: discharge home or rehabilitation facility Poor outcome: death or long-term nursing facility | Increase in incidence of good outcome in intervention group (21/43 vs. 9/34, p=0.046) |
|  | Hypothermia after Cardiac Arrest Study Group(57) | 2002 | Randomized controlled trial, with blinded outcome assessment | hypothermia vs. no temperature regulation | 275 | OHCA due to VF/pulseless VT | Median of 105 min (IQR 61-192) after ROSC | 24h | Partly | 32-34˚C vs. > 36.5˚C |  | Good outcome: CPC 1 or 2 at 6m Secondary: mortality at 6m, complication rate, bleeding | One in each group lost to follow-up. Increase in incidence of good outcome in intervention group (75/136 vs. 54/137, p=0.009)  No difference in complications, trend towards higher infection rates in hypothermia |
|  | Nielsen et al.(6) | 2013 | Randomized controlled trial, with blinded outcome assessment | hypothermia vs. strict normothermia | 950 | OHCA of presumed cardiac origin | <220 min after ROSC | 28h active cooling stops and gradual rewarming starts | Yes | 33˚C vs. 36˚C |  | Primary: all-cause mortality at 6m Secondary: poor neurologic outcome (CPC 3-5, mRS 4-6) | No differences in mortality or neurological outcome (mortality of 50% in hypothermia group vs. 48% in normothermia) Trend towards more serious adverse events in hypothermia (p=0.09) |
|  | Look et al.(60) | 2018 | Randomized controlled trial, open label | external vs. internal cooling vs. historic/ contemporary controls without TTM | 45 +  42 | OHCA of any cause (except trauma, intracranial haemorrhage) | median of 304.5 vs. 333 minutes | 24h followed by passively rewarming | Partly | 34˚C vs. 'normothermia' (not specified) |  | Primary: survival-to-hospital discharge and CPC at discharge between external vs. internal cooling Secondary: survival TTM vs. normothermia (historical controls) | No difference in survival or neurological outcome between external and internal cooling techniques Better survival in intervention group vs. normothermia controls 40% vs. 21.4% |
|  | Kirkegaard et al.(65) | 2017 | Randomized controlled trial, with blinded outcome assessment | 24h hypothermia vs. 48h hypothermia | 355 | OHCA of presumed cardiac origin | Median 281 (IQR 217-360) and 320 min (IQR 241-410) in resp 48h and 24h group | 48h hours | Yes | 33°C |  | CPC at 6 months (good outcome defined as CPC 1-2) Secondary: 6 month mortality and time to death | No improvements in 6 month neurological outcome |
|  | Arrich et al.(61) | 2016 | Cochrane review until march 2015 | pre-hospital cooling vs. in-hospital cooling | 2369 | OHCA of any cause (except one study with only witnessed collaps) | N/A | N/A | Yes | 32 to 34°C |  | Survival at 30 days and 6 months Neurological outcome by CPC (good outcome CPC 1-2) Secondary: adverse events | No evidence for a benefit of pre-hospital cooling vs. in-hospital cooling. With the sidenote that a relevant amount of participants did not receive pre- or in-hospital cooling for unknown reasons. |
|  | Scales et al.(62) | 2017 | Randomized controlled trial, open label | prehospital cooling postROSC vs. usual care | 585 | OHCA of any cause (except trauma, burn or exposure hypothermia) | Exact time unknown, but prehospital post ROSC | Hospital arrival | No | 32-34°C (only defined for prehospital) |  | Primary: successful temperature management defined as achieving 32-34°C within 6h after ED arrival Secondary: rates of applying TTM, neurological outcome by mRS scale (good 0-2) | No difference in rate of successful temperature management Larger change of in-hospital TTM applying in intervention group (68% vs. 56%, p=0.003) Similar survival with good neurological outcome No association with re-arrest during transport Decreased incidence of pulmonary edema (12% vs. 18%, p=0.04) |
|  | Nordberg et al.(63) | 2019 | Randomized controlled trial, open label | cooling during resuscitation vs. cooling after ROSC | 677 | Bystander witnessed OHCA of presumed cardiac origin | Exact time unknown, but prehospital intra-arrest | Hospital arrival | Yes | 33°C±1 |  | Primary: survival with good neurologic outcome (CPC 1-2) at 90 days Secondary: Overall survival at 90 days and cooling efficacy | No improvements in survival or good neurological outcome at 90 days. Time to target core temperature was shorter in intervention group (105 min vs. 182 min, p < 0.001) |
|  | Bernard et al.(64) | 2016 | Randomized controlled trial, open label | pre-hospital intra-arrest cooling via rapid cold saline infusion vs. in-hospital cooling | 1198 | OHCA of presumed cardiac origin | Exact time unknown, but prehospital intra-arrest | Hospital arrival | Partly | 33°C |  | Survival at discharge Secondary: ROSC, discharge to home | No differences in survival at hospital discharge (10.2% vs. 11.4%).  Decrease in gaining ROSC in shockable rhythms in intervention group (41.2% vs. 50.6%, p=0.03) No differences in discharge to home (8.7 vs. 8.4%) |
|  | Stöckl et al.(71) | 2017 | Randomized controlled trial, double blinded | Continuous NMB vs. placebo and on demand NMB | 65 | OHCA of presumed cardiac origin | Exact time unknown, but within 6 hours after ROSC | 29h | Yes | 33°C |  | Primary: number of shivering episodes during TTM Secondary: dose of rocuronium, survival, neurologic outcome (CPC) at 1 year | No differences in overall survival or neurological outcome Reduced shivering in intervention group (25% in continuous NMB vs. 94% in bolus NMB) |
|  | Lee et al.(72) | 2018 | Randomized controlled trial, open label | Continuous NMB vs. placebo with bolus NMB in intractable shivering | 85 | OHCA of presumed cardiac origin | Exact time unknown, but within 6 hours after ROSC | 24h | Yes | 33 or 36°C |  | Primary: serum lactate at 24h Secondary: poor neurological outcome at discharge (CPC 3-5 or mRS 4-6), in-hospital mortality | No difference in serum lactate levels at 24h No difference in poor neurologic outcome (76.3% vs. 76.7%, p=0.964) Placebo group required an average of three NMB infusions |
| *Decreasing cerebral metabolism* | | |  |  |  |  |  |  |  |  |  |  |  |
| **Barbiturates** | Brain Resuscitation Clinical Trial I study group(77) | 1986 | Randomized controlled trial, open label | Thiopental loading dose vs. standard therapy | 262 | Comatose after OHCA of any cause | 10-50 minutes after ROSC | N/A | No |  |  | CPC at 6 and 12 months, survival | No difference in survival (77% died in thiopental group vs. 80% in standard therapy) No difference in good outcome, (CPC 1-2 20% in thiopental vs. 15% in standard therapy) |
|  | Monsalve et al.(78) | 1987 | Non-randomized trial with matched controls | thiopental vs. historical cases | 53 +  54 | Comatose after OHCA of any cause | Within 30 minutes after ROSC | 24h | No |  |  | recovery of consciousness with normal motor and intellectual performance | No difference in survival (mortality of 58% in thiopental group vs. 70% in historical control) with a non-significant higher mortality rate in the first 6h in thiopental group.  But more neurologic recovery in thiopental group (61% vs. 37%, p<0.03) |
| *Glutamate antagonism* | | |  |  |  |  |  |  |  |  |  |  |  |
| **Xenon** | Laitio et al.(84) | 2016 | Randomized controlled trial, single blinded | inhaled xenon vs. standard care | 110 | Comatose after OHCA due to VF/VT | Median 247 min after OHCA | 18-34h after hospital admission | Yes | 33-34 ˚C |  | Primary: fractional anisotropy from diffusion tensor MRI  Secondary: CPC, mRS, mortality at 6 months | Higher global fractional anisotropy in xenon group (p=0.03) No difference in neurological outcomes (CPC 1 in both groups) or mortality at 6 months (survival of 27.3% in xenon group vs. 34.5% in control group) |
| **Exenatide** | Wiberg et al.(89) | 2016 | Randomized controlled trial, double blinded | exenatide vs. placebo | 118 | Comatose after OHCA of presumed cardiac origin | Median 162 minutes after ROSC | 6h15m | Yes | 36 ˚C |  | Feasibility and NSE from 24 to 72 hours. | Early administration (within 240 min) of exenatide in the ICU setting is feasible (>90% of cases).  No difference in median area under the NSE curve |
| **Scopolamine and penehyclidine hydrochloride** | Wang et al.(90) | 2018 | Randomized clinical trial, open label | scopolamine vs. penehyclidine hydrochloride | 80 | OHCA from acute myocardial infarction | ? | 24h | ? |  |  | ICP, cerebral oxygen partial pressure, CPP, NSE, cytokines, MRI, NIHSS | Higher CPP, lower ICP, NSE and NIHSS in penehyclidine hydrochloride groups compared to scopolamine |
| **Magnesium** | Hassan et al.(91) | 2002 | Randomized controlled trial, double blinded | magnesium vs. placebo | 105 | Refractory VF | Exact time unknown, but prehospital | N/A | No |  |  | Primary: ROSC with hospital discharge Secondary: admission to ICU, neurological outcome by GPOS | Three patients (2 received magnesium, 1 placebo) survived until hospital discharge, al had a good neurological outcome (GPOS 1) |
|  | Longstreth et al.(92) | 2002 | Randomized controlled trial, double blinded | magnesium and/or diazepam vs. placebo | 300 | Intubated after resuscitation from OHCA of any cause | Exact time unknown, but prehospital, minutes after ROSC | N/A | No |  |  | Primary: awakening at any time by 3 months. Secondary: days to awakening, days to death, independent at 3 months | No differences in 'awakening' or neurological outcome (defined as "independent") between magnesium, diazepam or combination vs. placebo |
| *Calcium antagonists* | |  |  |  |  |  |  |  |  |  |  |  |  |
| **Nimodipine** | Forsman et al.(95) | 1989 | Randomized controlled trial, double blinded | nimodipine vs. placebo | 51 | Comatose after OHCA due to VF or asystole (excl head trauma as a cause) | 21 minutes after ROSC | 10 hours | No |  |  | Primary: CBF, ICP, neurologic outcome measured by GCP-score | Greater CBF in nimodipine group, no difference in mortality (15 patients in nimodipine group vs. 16 in placebo were dead at 4 months) or in good neurological outcome (8 vs. 9 patients) |
|  | Roine et al.(94) | 1990 | Randomized controlled trial, double blinded | nimodipine vs. placebo | 155 | Witnessed OHCA due to VF (seen max 30 minutes after ROSC) | Mean 24 minutes after cardiac arrest | 24 hours | No |  |  | Primary: survival, outcome measured by glasgow outcome scale | No change in survival (40% in nimodipine group vs. 36% in placebo group) or neurological outcome (29 vs. 24% good neurological outcome) |
| **Lidoflazine** | Brain Resuscitation Clinical Trial II Study Group(97) | 1991 | Randomized controlled trial, double blinded | lidoflazine vs. placebo | 520 | Comatose after OHCA (seen max 30 minutes after ROSC | Mean 18 minutes after ROSC | 16 hours | No |  |  | Glasgow outcome scale, CPC, survival | No effect on survival (mortality 82% in lidoflazine vs. 83% in placebo) or good neurological outcome (14.7 vs. 12.9%) |
| *Preventing acidosis* | |  |  |  |  |  |  |  |  |  |  |  |  |
| **Sodium bicarbonate** | Ahn et al.(101) | 2018 | Randomized controlled trial, double blinded | bicarbonate vs. placebo | 50 | Non-traumatic OHCA with failed ROSC after 10 min + severe metabolic acidosis | Median 31 min after start CPR | N/A | ? |  |  | Primary: change in acidosis Secondary: sustained ROSC, survival to hospital admission, CPC 1 or 2 at 1 and 6 months | Higher pH in bicarbonate group (pH 6.99 vs. pH 6.9) No difference in sustained ROSC (3 in bicarbonate group vs. 6 patients in control group), hospital admission (1 vs. 4), good neurological outcome 1 month (0 vs. 1) and at 6 months (0 vs. 0) |
| *Anti-oxidants* | |  |  |  |  |  |  |  |  |  |  |  |  |
| **Oxygen** | Kuisma et al.(105) | 2006 | Randomized controlled trial, open label | 30% vs. 100% oxygen | 28 | OHCA | Exact time unknown, but immediately after ROSC | 60 minutes after start | Partly | Hypo- and normothermia |  | Primary: NSE, S-100 levels at 24 and 48h, the adequacy of oxygenation | Applying different oxygen regimens was feasible There were no differences in NSE levels (NSE at 48h 14.2 vs. 18.6 microg/L in 30% vs. 100% oxygen) |
|  | Jakkula et al.(135) | 2018 | Randomized controlled trial, open label | Normoxia (10–15 kPa) vs. moderate hyperoxia (20–25 kPa) | 123 | Comatose after witnessed OHCA due to VF/VT | median 166 minutes | 36 hours after ICU admission | Yes | 33 °C or 36 °C |  | Primary: NSE serum concentration at 48 h after cardiac arrest, feasibility Secondary: S100-b, troponin, regional frontal cerebral oxygenation, CPC at 6 months | Targeting normoxia or moderate hyperoxia was feasible, no effects on NSE at 48h (22.3 microg/L in normoxia vs. 20.6 micgrog/L in moderate hyperoxia) |
| **Sodium nitrite** | Kim et al.(113) | 2018 | Non-randomized trial with matched controls | Sodium nitrite 25mg and 60mg vs. matched controls | 120 | Comatose after cardiac arrest of non-traumatic origin | Exact time unknown, but minutes after standard ALS | N/A | Partly | 42-50% hypothermia |  | Primary: rate of ROSC, use of noradrenaline, first systolic blood pressure. Secondary: early haemodynamic effects, neurological outcome by CPC | 59 patients received 25 mg IV nitrite, because of too low serum levels, the following 61 received 60 mg IV nitrite.  No difference in ROSC (48% in 25mg and 49% in 60mg group vs. 59% in matched controls). No difference in CPC at discharge (good neurological outcome in 19% and 12% vs. 19%, p=0.55). No difference in noradrenaline use or first systolic blood pressure. |
| *Anti-inflammation* | |  |  |  |  |  |  |  |  |  |  |  |  |
| **Erythropoietin** | Cariou et al.(165) | 2008 | Non-randomized trial with matched controls | EPO vs. case matched historical controls | 18 + 40 | Comatose after witnessed OHCA of presumed cardiac origin (delay to CPR <10min, delay to ROSC <50min) | median 62 min | 48 hours after ICU admission | Yes | 32-34 °C |  | Primary: CPC day 1-7, 14, 21 and 28 | No difference between EPO-treated patients and matched controls at day 28 (CPC 1-4 55% versus 47.5%, p = 0.17). AE: one case of vascular arterial thrombosis |
|  | Grmec et al.(117) | 2009 | Non-randomized controlled trial with added matched controls | EPO vs. standard care vs. case matched controls | 54 + 48 | OHCA of non-traumatic cause | Exact time unknown, but prehospital, intra-arrest | N/A | Yes | 32-34 °C |  | Primary: ICU admission. Secondary: ROSC, survival at 24h, survival at hospital discharge | Higher ICU admission rates (92% in EPO vs. 50% in standard care group vs. 65% in matched controls) No difference in CPC at hospital discharge (good neurological outcome 69% vs. 67% vs. 73%). |
|  | Cariou et al.(166) | 2016 | Randomized controlled trial, single blinded | EPO vs. standard treatment | 476 | Comatose after witnessed OHCA of presumed cardiac origin and ROSC within 60min | Median time of 1.43 hours after ROSC | 48 hours | Partly | 32-34 °C (in 93.5%) |  | Primary: number of patients with CPC 1 at day 60 Secondary: distribution of CPC at day 30 and 60, mortality, adverse events | No difference in CPC 1 at day 60 (32.4% in EPO group vs. 32.1% in control group). No differences in CPC distribution between the groups. Comparable mortality rate (57.7% vs. 56.4%). AE: thrombotic complication in 12.4% of EPO group vs. 5.8% in control group. |
| **Glucocorticoid** | Tsai et al.(119) | 2007 | Non-randomized controlled, open labeled trial | hydrocortison vs. placebo | 97 | Non-traumatic OHCA without medical history of steroid use | Exact time unknown, but prehospital, intra-arrest | N/A | No |  |  | ROSC, sustained ROSC, survival at discharge, CPC at discharge | More patients gaining ROSC in hydrocortisone group (61% vs. 39%, p=0.038). No differences in CPC-scores (median CPC 4 in both groups in resp 3 vs. 6 surviving patients). |
| *Mitigating mitochondrial damage* | | |  |  |  |  |  |  |  |  |  |  |  |
| **Cyclosporine** | Argaud et al.(122) | 2016 | Randomized controlled trial, single blinded | Cyclosporine vs. standard care | 794 | Witnessed OHCA with non-shockable cardiac rhythm | Exact time unknown, but prehospital, intra-arrest | N/A | Partly | "hypothermia" in around 70% |  | Primary: multi organ failure by SOFA.  Secondary: survival at 24h, neurological outcome at discharge | No differences in median SOFA scores (10 in cyclosporine group vs. 11 in standard care group). No differences in survival at 24h (16.8 vs. 15.7%) or in good neurological outcome (1.8 vs. 1.3%) |
| **Coenzym Q10** | Damian et al.(125) | 2004 | Randomized controlled trial, blinded outcome assessment | CoQ10 vs. placebo | 49 | Comatose after witnessed cardiac arrest of presumed cardiac origin | mean 8.9 hours after cardiac arrest | ? (at least 24 hours) | Yes | 35-36 °C |  | Primary: survival to discharge from ICU, 3 month survival,  Secondary: GOS at 3 months, S100 at day 0,1 and 5 | Higher survival rate in CoQ10 group (68% vs. 29.2%). No difference in GOS (good neurological outcome 36% vs. 20%) |
| *Optimizing cerebral perfusion* | | |  |  |  |  |  |  |  |  |  |  |  |
| **Adrenaline** | Perkins et al.(129) | 2018 | Randomized controlled trial, double blinded | adrenaline vs. placebo | 8014 | OHCA (excl OHCA due to anaphylaxis or asthma) | Median 13,8 min after cardiac arrest | N/A | ? |  |  | Primary: survival at 30 days. Secondary: neurological outcome by mRS at hospital discharge | Higher survival rate at 30 days in adrenaline group (3.2% vs. 2.4%, p=0.02), but no difference in favourable neurological outcome between groups (2.2% vs. 1.9%) and more survivors with severe neurologic impairment in adrenaline group (31% vs. 17.8%) |
|  | Vargas et al.(163) | 2019 | Meta-analysis on RCT's until august 2018 | Standard dosed adrenaline vs. pooled treatments (placebo, no drugs, high dosed adrenaline, adrenaline + vasopressin) | 20716 | OHCA | Exact time unknown, but intra-arrest | N/A | ? |  |  | Primary: survival to hospital discharge Secondary: ROSC, survival to hospital admission, good neurological outcome | Improved survival to hospital discharge in standard dosed adrenaline compared to pooled treatments (RR 1.16, CI: 1.00-1.35, p=0.04) and to placebo/no drugs (RR 1.34, CI 1.08-1.67, p=0.00) Increased rate of good neurological outcome in standard dosed adrenaline vs. pooled treatments (RR 1.66, CI 1.00-1.35, p=0.04), but no differences compared to placebo/no drugs (RR 1.22, CI 0.99-1.52, p=0.06) |
| **Carbon dioxide** | Eastwood et al.(133) | 2016 | Randomized controlled trial, single blinded | normocapnia (35-45 mmHg) vs. mild hypercapnia (50-55 mmHg) | 83 | Non-traumatic OHCA (partly IHCA) receiving mechanical ventilation | Median 252 minutes from cardiac arrest to randomization | 24h after randomization | Yes | 33 °C or 36 °C |  | Primary: NSE and s100b in first 50 patients  Secondary: glasgow outcome scale extended (good ≥5) | Smaller increase in NSE in mild hypercapnia (p=0.04), no differences in s100b levels. No differences in good neurological outcome (59% in hypercapnia group vs. 46% in normocapnia, p=0.26) |
|  | Jakkula et al.(135) | 2018 | Randomized controlled trial, open label | low normal PaCO2 (33-35 mmHg) vs. high normal PaCO2 (43-45 mmHg | 123 | Comatose after witnessed OHCA due to VF/VT | Median 159 minutes from ROSC to randomization | 36 hours after ICU admission | Yes | 33 °C or 36 °C |  | Primary: NSE serum concentration at 48 h after cardiac arrest, feasibility Secondary: S100-b, troponin, regional frontal cerebral oxygenation, CPC at 6 months | No difference in NSE at 48 h (18.8 microg/L in low-normal vs. 22.5 microg/L in high-normal PaCO2, p=0.400) No difference in rate of good neurological outcome at 6 months (71% vs. 59%, p=0.200) |
| **Mean arterial pressure** | Jakkula et al.(138) | 2018 | Randomized controlled trial, open label | low-normal (65-75 mmHg) vs. high-normal (80-100 mmHg) MAP | 120 | Comatose after witnessed OHCA due to VF/VT | Median 171 minutes from ROSC to randomization | 36 hours after ICU admission | Yes | 33 °C or 36 °C |  | Primary: NSE serum concentration at 48 h after cardiac arrest, feasibility Secondary: S100-b, troponin, regional frontal cerebral oxygenation, CPC at 6 months | No difference in NSE at 48 h (20.6 microg/L in low-normal vs. 22.0 microg/L in high normal MAP, p=0.522). No differences in rate of good neurological outcome at 6 months (62 vs. 68%, p=0.444) |
|  | Ameloot et al.(139) | 2019 | Randomized controlled trial, open label with blinded outcome assessment | EGDHO (MAP 85-100 mmHg, SvO2 65-75%) vs. standard care (MAP 65 mmHg) | 112 | Comatose after OHCA of presumed cardiac cause (excl intracranial hemmorrhage/stroke) | Exact time unknown, but after ICU admission | 36 hours after ICU admission | Yes | 33 °C |  | Primary: extent of anoxic brain damage quantified as percentage of ischaemic voxels on MRI-DWI Secondary: favourable neurological outcome at ICU discharge and 180 days by CPC | No different percentage of ischaemic voxels on MR-DWI (median 16% in EGDHO vs. 12% in standard care, p=0.09) and it was a poor predictor of good neurological outcome. No differences in good neurological outcome at ICU discharge (43% vs. 27%, p=0.15) |
| **Thrombolysis** | Abu-Laban et al.(143) | 2002 | Randomized controlled trial, double blinded | t-PA (activase) vs. placebo | 233 | PEA > 1 minute and no pulse after CPR > 3 minutes | Intra-arrest, mean of 36 minutes (median 35) after cardiac arrest | N/A (CPR at least continued for 15 minutes) | No |  |  | Primary: survival to hospital discharge Secondary: ROSC, length of hospital stay, haemorrhage and neurological outcome by GCS | No differences in survival to hospital (6.0% in t-PA vs. 5.2 % in placebo group, p=0.99), only 1 patient survived to hospital discharge. Major haemorrhage occurred in 1.7% in t-PA vs. 0 in placebo. |
|  | Böttiger et al.(144) | 2008 | Randomized controlled trial, double blinded | Tenecteplase vs. placebo | 1050 | Witnessed OHCA of presumed cardiac origin and start BLS <10 min | Prehospital, median of 18 minutes after cardiac arrest | N/A (CPR at least continued for 30 minutes) | No |  |  | Primary: 30 day survival Secondary: hospital admission, ROSC, neurological outcome by CPC | No differences in 30 day survival (14.7% in tenecteplase group vs. 17.0% in placebo group, p=0.36). No differences in hospital admission, ROSC or neurological outcome (CPC 1 in 41 vs. 45 patients, RR 1.02, CI 0.75-1.38). Intracranial haemorrhage occurred with greater frequency in tenecteplase group (2.7% vs. 0.4%, p=0.006) |
| *Supportive therapies* | |  |  |  |  |  |  |  |  |  |  |  |  |
| **Sedation** | Bjelland et al.(145) | 2012 | Randomized controlled trial, open label | propofol/remifentanyl vs. midazolam/fentanyl | 59 | Patients receiving therapeutic hypothermia after cardiac arrest | Exact time unknown, but at ICU admission | not described | Yes | 34 °C |  | Primary: time to either extubation/decision that extubation was clinically undesirable.  Secondary: CPC | Shorter time to offset in propofol/ remifentanil group (13.2 h vs. 36.8 h, p<0.001 between discontinuation and extubation). No difference in neurological outcome |
|  | Paul et al.(146) | 2018 | prospective propensity-matched cohort study | midazolam/fentanyl (2008 to 2013) vs. propofol/remifentanyl (2014 to 2016) | 326 vs. 134 | Comatose after OHCA | Exact time unknown, but at ICU admission | 34-40h | Yes | 32-34 °C |  | Primary: delayed awakening (persisting unconsciousness 48h after discontinuation of sedation) Secondary: rate of seizure, self-extubation, CPC | Less delayed awaking in propofol/remifentanyl (29% vs. 6%, p<0.001). No difference in good neurological outcome (94% vs. 92% of surviving patients, p=0.50) |
| **Glucose** | Oksanen et al.(148) | 2007 | Randomized controlled trial, open label | strict glucose control (4-6 mmol/L) vs. moderate control (6-8 mmol/L) | 90 | Comatose after OHCA due to VF of presumed cardiac origin (ROSC<35 min) | Exact time unknown, but within 4 hours after ROSC | 48 h | Yes | 33 °C |  | Primary: mortality at 30 days Secondary: NSE at 24 and 48h | No difference in mortality at 30 days (33% in strict vs. 35% in moderate glucose control, p=0.846) Not powered for detecting differences in NSE-levels and no significant differences at 24 and 48 h |
| **Prophylactic antibiotics** | Ribaric et al.(149) | 2017 | Randomized controlled trial, open label | immediate prophylactic amoxicillin-clavulanic acid vs. clinically-driven antibiotics | 60 (+ 23 in  registry) | Comatose after OHCA of presumed cardiac origin (patients with aspiration on admission were excluded and registered) | Exact time unknown, but after bronchoscopy performed after ICU admission | 7 days | Yes | 32-34 °C |  | Primary: severity of SIRS (estimated by white blood count, C-reactive protein, procalcitonine, CD 64) Secondary: pneumonia on chest X-ray, ICU survival, good neurological outcome  ALS indicates Advances Life Support; CPC, Cerebral Performance Category; CPR, cardiopulmonary resuscitation; EGDHO, early goal-directed haemodynamic optimization strategy; EPO, erythropoietin; GCS, Glasgow coma scale; ICU, intensive care unit; IHCA, in hospital cardiac arrest; MAP, mean arterial pressure; mRS, modified Rankin Scale; N/A, not applicable; NSE, neuron specific enolase; OHCA, out of hospital cardiac arrest; RCT, randomized controlled trial; ROSC, return of spontaneous circulation; SIRS, systemic inflammatory response syndrome; TTM, targeted temperature management; VF, ventricular fibrillation; VT, ventricular tachycardia | Proportion of patients on antibiotics was greater in prophylactic group between day 1 and 5 and not on day 6 and 7.  No major impact on SIRS. No difference in good neurological outcome (50% in prophylactic antibiotics vs. 40% in clinically-driven antibiotics, p=0.60) |
